# Risks associated with oral deferiprone in the treatment of infratentorial superficial siderosis

**DOI:** 10.1007/s00415-019-09577-6

**Published:** 2019-10-16

**Authors:** Y. Sammaraiee, G. Banerjee, S. Farmer, B. Hylton, P. Cowley, P. Eleftheriou, J. Porter, D. J. Werring

**Affiliations:** 1 Stroke Research Centre, Department of Brain Repair and Rehabilitation, UCL Institute of Neurology, The National Hospital for Neurology and Neurosurgery, Russell Square House, London, WC1N 5EE UK; 2 National Hospital for Neurology and Neurosurgery, London, UK; 3 Department of Haematology, University College London, London, UK

**Keywords:** Superficial siderosis, Deferiprone, Dural defects, Iron chelation, Agranulocytosis

## Abstract

**Objective:**

Deferiprone is an iron chelator that has recently been used to treat patients with infratentorial superficial siderosis (iSS). It is considered to have a generally favourable safety profile but concerns have been raised due to the risk of agranulocytosis. We aimed to evaluate the safety and tolerability of oral deferiprone as a treatment for patients with iSS.

**Methods:**

We present a case series of 10 consecutive patients presenting with classical iSS treated with deferiprone.

**Results:**

Ten patients were followed up for a mean period of 2.3 years (range 0.5–5.5 years). Four patients (40%) were withdrawn from treatment because of treatment-related side effects. The reasons for treatment discontinuation were neutropenic sepsis (*n* = 3) and fatigue (*n* = 1). In 2 out of the 3 cases of neutropenic sepsis, patients initially developed neutropenia without sepsis. The mean time to neutropenic sepsis following deferiprone was 1.2 years (range 0.3–2.5) with mean neutrophil count of 0.4 (range 0.3–0.5). Six patients (60%) reported no change in neurological function while on treatment, and four patients (40%) reported that their condition deteriorated.

**Conclusions:**

Deferiprone was poorly tolerated, with 40% of patients withdrawing from treatment, most commonly due to neutropenic sepsis, after an average of 2 years on treatment. This study increases the number of reported cases of agranulocytosis in patients with iSS treated with deferiprone. Clinicians treating iSS patients with deferiprone should be aware that this drug has a potentially life-threatening side effect of neutropenic sepsis, and should ensure that appropriate haematological monitoring is in place.

## Introduction

**Fig. 1:**
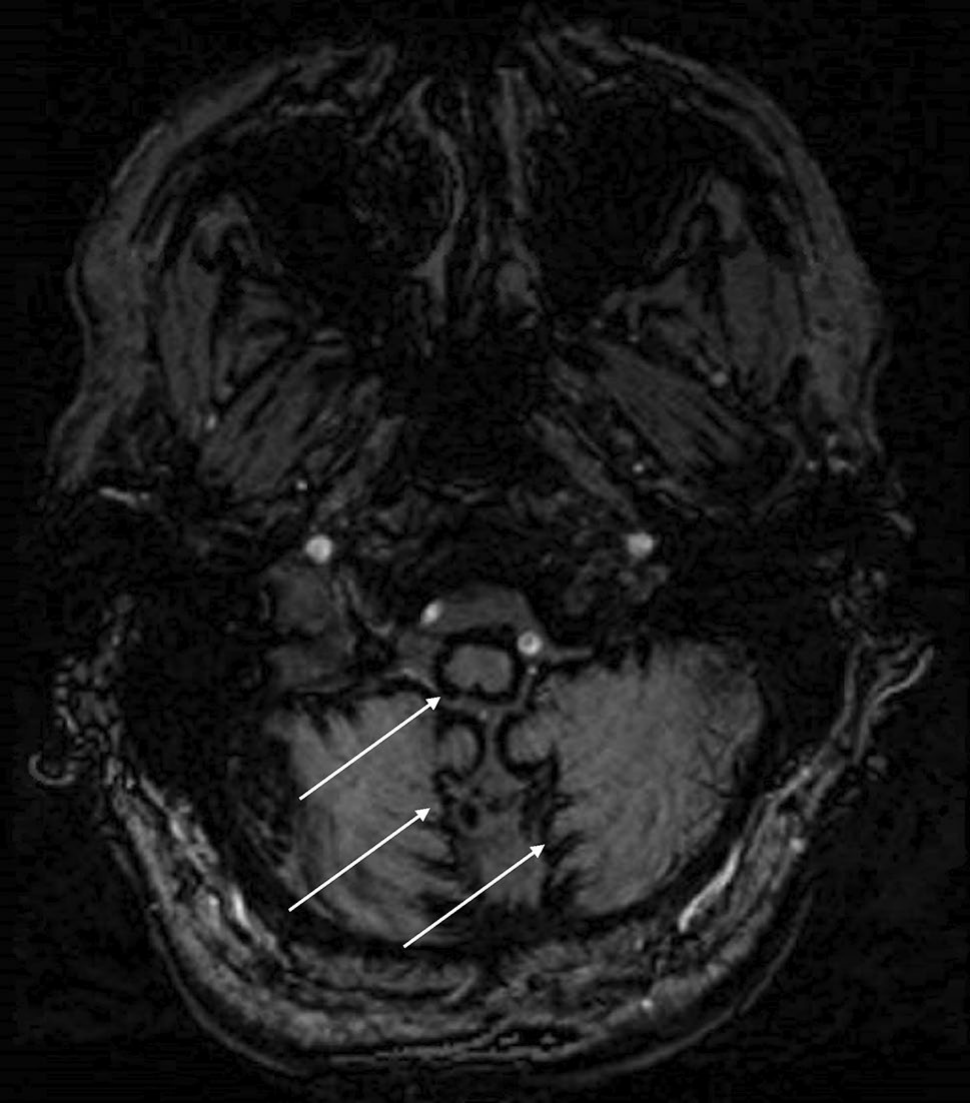
Type 1 (classical) infratentorial superficial siderosis shown on Susceptibility weighted Imaging (SWI). White arrows indicate areas of haemosiderin deposition

Central nervous system (CNS) infratentorial superficial siderosis (iSS) is a rare but disabling neurological condition that results from hemosiderin deposition on the pial surface of the brain and spinal cord [1]. Classical iSS has been previously defined as a distinct group of patients who have typical radiological features of superficial siderosis (Fig. 1) in the absence of any obvious intracranial bleeding event, and associated with a slowly progressive neurological deterioration (usually a combination of deafness, ataxia and myelopathy) [2]. It is hypothesised to result from a chronic slow and low-volume leak of red blood cells into the subarachnoid space [3]. The most common causes of iSS are dural defects, usually due to trauma or previous surgical procedures [4].

No treatment has yet been proven to alter the disease course of iSS [4]. Iron chelation therapy with deferiprone (initially developed for treatment of thalassaemia), which readily crosses the blood brain barrier, has been used to treat patients with iSS but its efficacy remains unproven [2, 5]. Deferiprone is considered to have a generally favourable safety profile [2], but concerns have been raised because of a single case report of agranulocytosis [6]. It is currently not known whether this risk is greater in patients without systemic iron overload, such as those with iSS, than in patients with transfusional iron overload where the risk is about 1% [7].

## Methods

This is a case series of consecutive patients presenting with classical iSS who were treated with deferiprone at the National Hospital for Neurology and Neurosurgery, Queen Square, London (a tertiary neurology centre). The project involved a retrospective review of routinely collected clinical data from March 2013 until January 2019, and was approved as a clinical service evaluation by the Clinical Governance department of the National Hospital for Neurology and Neurosurgery.

All patients were reviewed by a professor of vascular neurology (DJW) or a consultant neurologist (SF), both with expertise in iSS. Patients underwent a standardised series of investigations [4] including brain and spinal MRI, CSF analysis and CT myelography (as appropriate), all of which were reviewed by a multi-disciplinary team (MDT) of neurologists, neuroradiologists, neurosurgeons, and haematologists, with input from clinical biochemists. If the MDT was unable to identify a cause treatable via radiological or surgical means, then the patient was considered for medical treatment with deferiprone.

Patients were started on oral deferiprone, usually at a dose of 500 mg twice daily and up-titrated to 30 mg/kg, as suggested in a previous safety trial [5]. Monitoring included regular full blood counts (initially weekly), and serum ferritin (measured every 4–6 weeks). If stable after 3 months on treatment, a full blood count was then performed every 4 weeks. Changes in patient-reported neurological function and side effects were collected by a neurologist during clinic visits every 3–6 months. Our outcomes of interest were: agranulocytosis (absolute neutrophil count of 0.5 × 10^9^/L or lower), neutropenic sepsis (temperature higher than 38 °C or symptoms and/or signs of sepsis in a person with a neutrophil count of 0.5 × 10^9^/L or lower) and neutropenia (absolute neutrophil count of 2.0 × 10^9^/L or lower).

MRI T2*-weighted gradient-echo (GRE) and Susceptibility Weighted Imaging (SWI) scans were performed at baseline and yearly following initiation of treatment. There are no current validated scales to measure the extent of siderosis burden in patients with iSS. MRI images were subjectively reviewed by a senior neuroradiologist to assess whether siderosis was focal or extensive as well as to monitor for changes in infratentorial siderosis burden over time. Despite the lack of scales available to assess clinical severity, we subjectively classified patient’s disease severity as ‘Severe’ if patient’s symptoms impair their ability to lead an independent life. Specifically, patients with either bilateral hearing loss OR ataxia requiring a walking aid were classed as having ‘Severe’ disease.

## Results

Ten patients were included during this period (Table 1). The average age was 52 years (range 29–71 years), and 70% (*n* = 7) were male. Hearing impairment and coordination dysfunction were present in all patients, and two patients (20%) had clinical evidence of myelopathy. A dural defect associated with neurosurgery or trauma was present in five patients and CNS tumour/cyst was present in three patients. Two patients underwent surgical intervention for their iSS prior to starting deferiprone. One patient was found to have incidental neutropenia of unknown cause and was concurrently started on granulocyte colony stimulating factor (GCSF) prophylactically.

**Table 1:** Patient demographics

Mean age (years) (SD)	52 (12)
Sex	
Male	7 (70%)
Female	3 (30%)
Ethnicity	
White	9
Chinese	1
Median disease duration (years) (IQR)	5.5 (4.5)
Presumed aetiology	
Dural defect	5
CNS tumour/cyst	3
Ankylosing spondylitis	1
Undetermined	1
Surgical repair of dural defect	2 (20%)

The average duration on treatment was 2.3 years (0.5–5.5 years). Individual patient outcomes are shown in Table 2. Four patients (40%) were withdrawn from treatment because of treatment-related side effects (mean time on treatment 2.1 years, range 0.5–4 years). The reasons for treatment discontinuation were neutropenic sepsis (*n* = 3) and fatigue (*n* = 1). In 2 out of the 3 cases of neutropenic sepsis, patients initially developed neutropenia without sepsis. One patient temporarily stopped deferiprone treatment and the other continued treatment but was concurrently started on GCSF. No other patient started GCSF during the study period. Both patients had documented recovery of neutrophil count after 2 and 3 months, respectively. They both later developed neutropenic sepsis while on deferiprone, after which treatment was permanently stopped. The mean time to neutropenic sepsis following deferiprone was 1.2 years (range 0.3–2.5) with a mean neutrophil count of 0.4 (range 0.3–0.5). One patient stopped treatment after 1 year following complications relating to a pre-existing brain tumour, and subsequently died (within 6 months of cessation). One patient developed dose-dependent arthralgia in both knees but continued treatment.

**Table 2:** Outcomes and side effect profile of Superficial Siderosis patients treated with deferiprone

No	Age	Sex	Cause of SS	Clinical features			Clinical severity	Initial dosing strategy (mg/kg/day)	Treatment duration (years)	Subjective change in symptoms	Baseline imaging severity	Follow-up MRI (years)	Significant side effect (s)
				Deafness	Ataxia	Myelopathy							
1	40	M	CNS tumor/cyst	Yes	Yes	No	Severe	24	4	Same	Extensive	Stable (3)	Fatigue*
2	52	M	Dural defect	Yes	Yes	No	Severe	10	2	Same	Extensive	Stable (2)	None
3	51	M	CNS tumor/cyst	Yes	Yes	No	Severe	15	2.5	Same	Extensive	Stable (2)	None
4	52	M	CNS tumor/cyst	Yes	Yes	No	Severe	12.5	1	Same	Extensive	Stable (1)	None
5	63	M	Dural defect	Yes	Yes	No	Severe	15	0.5	Same	Extensive	N/A	Neutropenic sepsis*
6	42	F	Dural defect	Yes	Yes	No	Moderate	15	1	Worse	Extensive	Stable (1)	None
7	71	M	Ankylosing spondylitis	Yes	Yes	No	Severe	18	3	Worse	Extensive	Stable (1)	None
8	52	F	Undetermined	Yes	Yes	Yes	Severe	27	5.5	Worse	N/A**	N/A	Joint pain
9	29	M	Dural defect	Yes	Yes	Yes	Severe	25	3	Worse	Extensive	Stable (3)	Neutropenia, neutropenic sepsis*†
10	67	M	Dural defect	Yes	Yes	No	Severe	30	1	Same	Extensive	Stable (1)	Neutropenia, neutropenic sepsis*

MRI T2*-weighted gradient-echo (GRE) and Susceptibility Weighted Imaging (SWI) scans performed at baseline and at latest yearly interval were subjectively analysed to ascertain whether siderosis was focal or extensive and whether there was evidence of improvement or progression over time

^*^Patient withdrew from treatment

^†^Incidental neutropenia of unknown cause—concurrently treated with GCSF

^**^ MRI not performed due to cochlear implant

Four patients had at least one recorded measurement of low serum ferritin (<30 µg/L), and one patient started iron replacement therapy. Two of these patients discontinued treatment (one due to fatigue and the other following episodes of neutropenic sepsis). Six patients (60%) reported no change in neurological function while on treatment, and four patients (40%) reported that their condition deteriorated. There was no evidence of initial transitory improvement in the first 3–6 months following starting treatment.

Nine patients (90%) underwent MRI imaging at baseline (Table 2) and all were found to have evidence of extensive infratentorial SS. On the latest yearly follow-up scans, 8 patients had scans available (mean time from start of treatment 1.75 years), all of which showed no significant change in siderosis burden following treatment.

## Discussion

In this case series of patients with infratentorial superficial siderosis, deferiprone was poorly tolerated, with 40% of patients withdrawing from treatment, most commonly due to neutropenic sepsis after an average of 2 years on treatment.

This study increases the number of reported cases of complications related to neutropenia in patients with iSS treated with deferiprone; we are aware of only one previously reported case of agranulocytosis [6]. The largest study of deferiprone in iSS (*n* = 38) reported no episodes of agranulocytosis during a 2 year follow-up period [2]. Interestingly, in the previous study, patients who developed neutropenia were not re-challenged with deferiprone following discontinuation of drug due to concerns about further side effects. It is possible that the increased rate of agranulocytosis in cohort is due to two of our patients being re-challenged following development of neutropenia and subsequently developing agranulocytosis. It may be the case that these particular patients are more susceptible to neutropenia for patient-specific reasons, and that re-challenge resulted in a more severe reaction. There were no other significant differences in baseline characteristics (including disease duration) in our study compared to the previous report [2]. Furthermore, all cases of agranulocytosis in our study occurred within 2.5 years of treatment initiation, broadly similar to the follow period reported in the above study, making it unlikely that there is an increased risk of neutropenia with longer treatment duration.

The mechanism of agranulocytosis with deferiprone is unclear. It has been argued that the effect is idiosyncratic or immune-mediated [7] and independent of dose or of iron overload level [7], although these variables were not studied systematically. However, deferiprone causes dose-dependent neutropaenia and bone marrow hypoplasia in laboratory animals, inhibiting granulocyte/macrophage colony forming units (CFU-G + Mac) in a dose-dependent manner, as well as causing cell cycle arrest through inhibition of ribonucleotide reductase [8]. Increased toxicity of deferiprone was previously reported in mice lacking iron overload than in overloaded animals [9]. Agranulocytosis was also noted in two out of 20 (10%) of patients receiving deferiprone receiving treatment for Parkinson's disease for 6 months [10]; these patients also lacked systemic iron overload. In the absence of generalised iron overload, relatively more iron-free chelator will be available in the systemic circulation for chelation of key metabolic iron pools involved in cell division. Thus, the increased agranulocytosis risk (30%) in this series is consistent with such a chelation-mediated mechanism.

In our cohort, 60% of patients reported stability of their neurological symptoms. Previously published data described 63% of subjects reporting no progression of disease or an improvement in at least one neurological domain over a period of 2 years on deferiprone [2]. iSS is known to lead to progressive neurological decline, but periods of stability may arise in the natural history of the disease [3]. It was clear that there was no detectable clinical or radiological response to deferiprone in our cohort, even for those who tolerated the drug for a reasonable period of time. The advanced degree of the disease, as evidenced by the clinical and radiological findings in these patients, could partly explain the lack of success of this treatment. We also acknowledge the lack of validated scores to quantify the progression of impairments and functional ability in patients with iSS.

Despite the retrospective case-based nature of the study and absence of long-term follow-up, we have shown that deferiprone can be associated with significant side effects in the treatment of iSS. The limitations in our study reflect the fact that iSS is a rare disease, without consensus criteria for investigating clinical and radiological progression. An international multicentre randomised controlled trial with clearly defined clinical and surrogate endpoints using validated scales and biomarkers would be challenging given the rarity of iSS, but could help to establish the hazards, benefits and effects on disease pathophysiology following deferiprone treatment. In the meantime, clinicians treating iSS patients with deferiprone should be aware that treatment has potentially life-threatening side effects, and ensure that appropriate monitoring is in place so that these events can be rapidly identified and treated.
